# Changes in the Harpagide, Harpagoside, and Verbascoside Content of Field Grown *Scrophularia lanceolata* and *Scrophularia marilandica* in Response to Season and Shade

**DOI:** 10.3390/metabo11070464

**Published:** 2021-07-19

**Authors:** Korey J. Brownstein, Andrew L. Thomas, Hien T. T. Nguyen, David R. Gang, William R. Folk

**Affiliations:** 1 Institute of Biological Chemistry, Washington State University, Pullman, WA 99164, USA; 2 Southwest Research Center, Division of Plant Sciences, University of Missouri, Mt. Vernon, MO 65712, USA; ThomasAL@missouri.edu; 3 Department of Anthropology, Washington State University, Pullman, WA 99164, USA; thuhienclrri@gmail.com; 4 Department of Biochemistry, University of Missouri, Columbia, MO 65211, USA

**Keywords:** abiotic stress, antioxidant, harpagide, harpagoside, *Scrophularia*, verbascoside

## Abstract

*Scrophularia lanceolata* Pursh and *Scrophularia marilandica* L. are two common species within the Scrophulariaceae family that are endemic to North America. Historically, these species were used by indigenous peoples and colonialists to treat sunburn, sunstroke, frostbite, edema, as well as for blood purification, and in women’s health. Several iridoid and phenylethanoid/phenylpropanoid glycosides detected in these species, such as harpagoside and verbascoside, possess anti-inflammatory and anti-nociceptive properties. Due to the presence of anti-inflammatory metabolites and the historical uses of these species, we performed a two-year field study to determine the optimal production of these important compounds. We subjected the plants to shade treatment and analyzed differences in the metabolite composition between the two species and each of their leaves, stems, and roots at various times throughout the growing seasons. We determined that *S. lanceolata* plants grown in full sun produced 0.63% harpagoside per dried weight in their leaves compared to shade-grown plants (0.43%). Furthermore, *S. lanceolata* accumulated more harpagoside than *S. marilandica* (0.24%). We also found that verbascoside accumulated in the leaves of *S. lanceolata* and *S. marilandica* as the growing season progressed, while the production of this metabolite remained mostly seasonally unchanged in the roots of both species.

## 1. Introduction

*Scrophularia* L. (Scrophulariaceae family) comprises about 200 species found primarily in the Northern Hemisphere, with some as far south as the Tropic of Cancer [1]. Many species in this genus are used in traditional, complementary, and alternative medicines to treat pain and inflammation [2,3,4]. Of the perennial North American *Scrophularia* species, *S. lanceolata* Pursh, one of the most common, is native to the northern two thirds of the United States into southern Canada, whereas *S. marilandica* L. is native to eastern North America. They are typically associated with damp, partly shaded areas near the edge of woodlands, stream banks, and bottomland forests, but are also found growing in full sun. *S. lanceolata* is tolerant of considerable habitat disturbance and can be considered “weedy” (personal communication with weed identification specialist, Richard Old). These species have a history of medicinal use by indigenous peoples and colonialists [5,6,7]. They are also easily cultivated [8].

*Harpagophytum procumbens* DC. ex Meisn., a plant in the Pedaliaceae family, is an important traditional medicine plant of sub-Saharan Africa used by the San, Khoi, and Bantu peoples [9]. It is a slow-growing, desert-adapted, perennial tuberous plant with fruits that have numerous long hooked spines, giving rise to the colloquial name, devil’s claw. Secondary tubers are ground or processed into extracts used throughout Africa, Europe, and the Americas for treatment of musculoskeletal, inflammatory, and other health problems that cause pain, loss of function, and disability [10,11,12,13]. While some efforts have been made to domesticate *H. procumbens* (e.g., Schneider, et al. [14]), it remains undeveloped as a cultivated crop and is challenging to grow. Because of the high value and enormous popularity of this species as a dietary supplement, natural populations are being over-harvested and even decimated [9]. Discovering alternative, easily cultivated species that produce significant amounts of similar anti-inflammatory metabolites may benefit many with such conditions and help protect *H. procumbens* from overexploitation.

As with *H. procumbens*, metabolite analysis of several endemic North American *Scrophularia* species suggests the traditional use of these plants for pain and inflammation may be due to their iridoid and phenylethanoid/phenylpropanoid glycoside content [15]. This includes the metabolite harpagoside (found in *H. procumbens*, as well as both *S. lanceolata* and *S. marilandica*), which has been shown to possess anti-inflammatory and anti-nociceptive properties [2,3,4,16,17,18,19,20]. While harpagoside is thought to be primarily responsible for the bioactivity of *H. procumbens*, two other anti-inflammatory metabolites, verbascoside (synonyms: acteoside and kusaginin) and harpagide, have been detected in this species (reviewed in Mncwangi, et al. [11]). These metabolites have also been identified in *S. lanceolata* and *S. marilandica* [15].

For these *Scrophularia* species to be cultivated as economic alternatives to *H. procumbens*, it is essential to understand the optimal production of harpagoside and metabolites with similar bioactivity under various field conditions. Other than harpagoside [21,22,23,24,25,26,27,28], little is known about how phenylethanoid/phenylpropanoid and other iridoid glycosides are affected by environmental inputs. To provide insights into the seasonal variation of other iridoid glycosides, Brownstein, et al. [8] analyzed the metabolite composition of field-cultivated *S. lanceolata* and *S. marilandica* in southwest Missouri. That study found harpagoside accumulated more abundantly in *S. lanceolata* leaves. Brownstein, et al. [8] also found that harpagide accumulated in the stems of *S. lanceolata* and *S. marilandica*, and these results were comparable to growth chamber plants [15]. Here, we present additional findings on *S. lanceolata* and *S. marilandica* plants grown in the field from 2014 to 2015.

## 2. Results and Discussion

**Figure 1:**
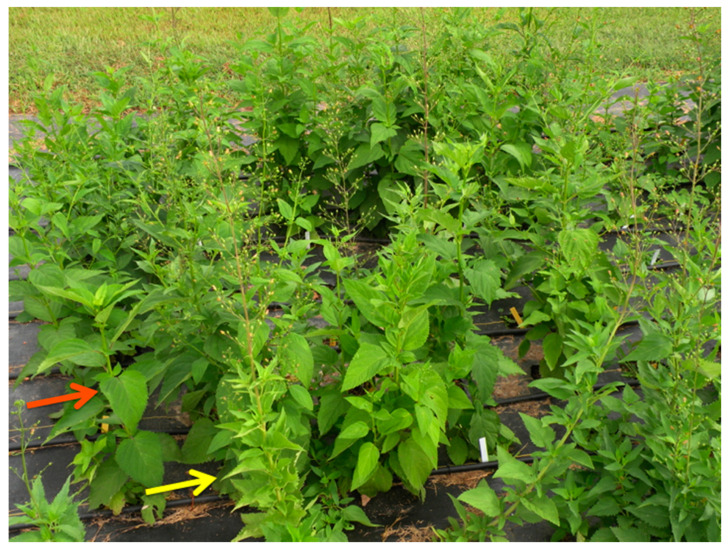
Morphological and phenological differences between *S. lanceolata* (yellow arrow) and *S. marilandica* (orange arrow) plants grown in the field.

**Figure 2:**
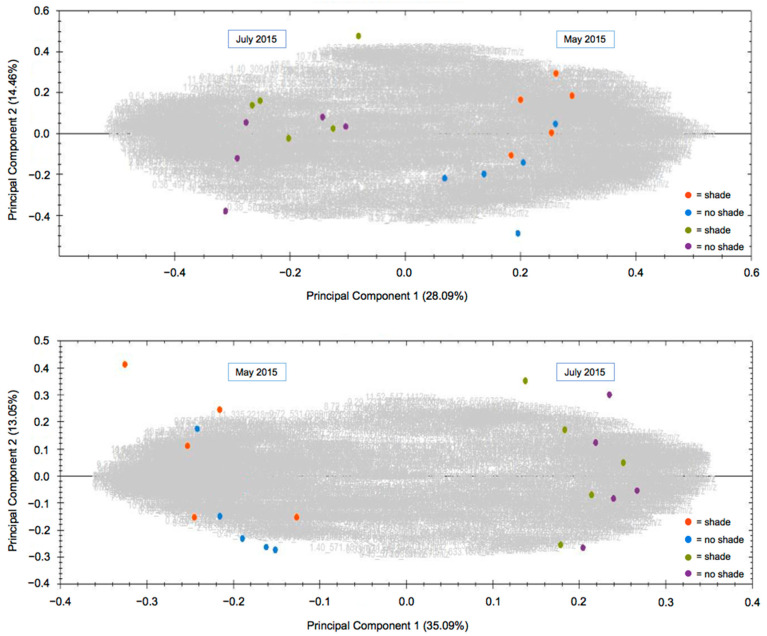
Principal component analysis (PCA) comparing leaves from no shade and shade-grown *S. lanceolata* (**top**) and *S. marilandica* (**bottom**) grown during May 2015 and July 2015. Each dot represents a biological replicate, and the grayed-out numbers are individual mass spectral features.

**Figure 3:**
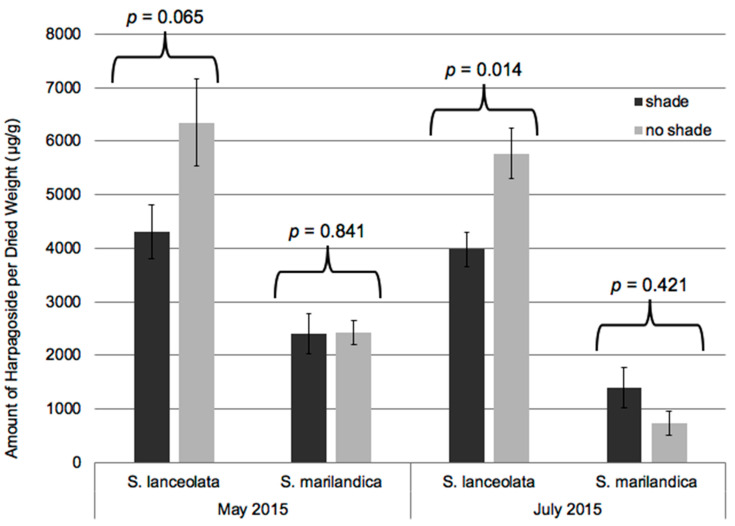
UPLC-PDA quantification of harpagoside content in the leaves of *S. lanceolata* and *S. marilandica* plants subjected to no shade and shade treatment that were harvested in May 2015 and July 2015. The vertical error bars represent standard errors of five biological replicates (n = 5). Statistical significance (*p* ≤ 0.05) was determined by independent/unpaired two-sample *t*-test. Since the Shapiro–Wilk test determined that the *S. marilandica* groups were not normally distributed, *p*-values for these groups were calculated by the Wilcoxon test.

**Figure 4:**
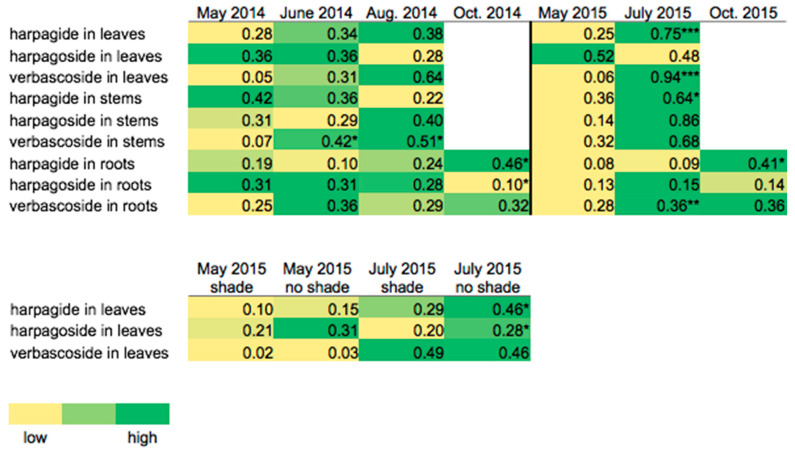
Heatmaps comparing relative abundances (low [0.00] to high [1.00]) of harpagide, harpagoside, and verbascoside in *S. lanceolata* tissues (leaves, stems, and roots) over the growing season and under shade and no shade treatments. For the October 2014 and October 2015 harvests, the plants had mostly senesced for the season; therefore, only the roots were collected. The vertical black line between the 2014 and 2015 seasons indicates that the seasons are not continuous. Statistical significance (*p* ≤ 0.05) was determined by independent/unpaired two-sample *t*-test or Wilcoxon test. *, significant at *p* ≤ 0.05; **, significant at *p* ≤ 0.01; and ***, significant at *p* ≤ 0.001. Figure S1 shows a bar chart version of this figure.

**Figure 5:**
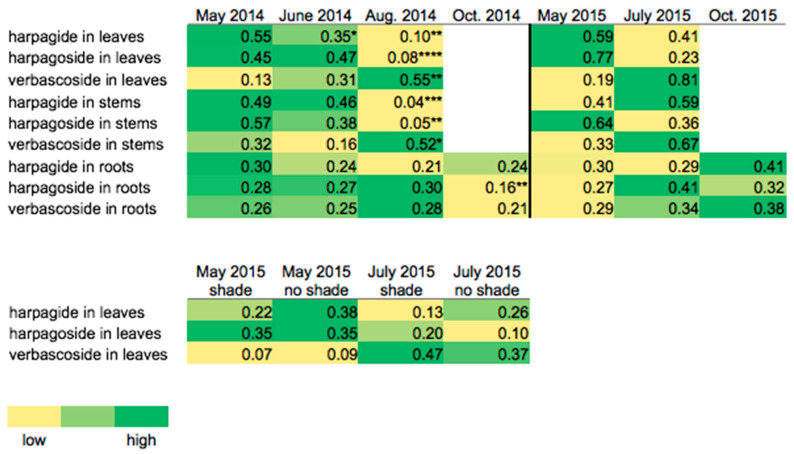
Heatmaps comparing relative abundances (low [0.00] to high [1.00]) of harpagide, harpagoside, and verbascoside in *S. marilandica* tissues (leaves, stems, and roots) over the growing season and under shade and no shade treatments. For the October 2014 and October 2015 harvests, the plants had mostly senesced for the season; therefore, only the roots were collected. The vertical black line between the 2014 and 2015 seasons indicates that the seasons are not continuous. Statistical significance (*p* ≤ 0.05) was determined by independent/unpaired two-sample *t*-test or Wilcoxon test. *, significant at *p* ≤ 0.05; **, significant at *p* ≤ 0.01; ***, significant at *p* ≤ 0.001; and ****, significant at *p* ≤ 0.0001. Figure S2 shows a bar chart version of this figure.

The plants established and grew well, with approximately 85% survival and most plants maturing and flowering the year after establishment. Periodic short-term summer droughts were mitigated by irrigation as needed so that drought stress should not have been a factor in the study. Temperatures during the study were typical of Southwest Missouri (hot summers and cold winters) with only one period of heat (23–25 August 2014; 38.1 °C) that would be classified as “extreme” [29]. Growing Degree Day data indicated that the 2014 and 2015 growing seasons were 0.7% and 3.4% warmer than the 30-year average, respectively (Table S1). Morphological and phenological differences between the two species described in Brownstein, et al. [8] remained consistent during the subsequent years of this study, with *S. marilandica* growing significantly taller (1.91 m) compared to *S. lanceolata* (1.18 m; *p* ≤ 0.0001), but inflorescence height and stem number were not statistically different. As shown in Figure 1, *S. lanceolata* also flowered earlier than *S. marilandica*, concurrent with Brownstein, et al. [8]. Samples of roots harvested and dried at the conclusion of the 2014 growing season (October 2014) indicated that newly dormant roots of *S. lanceolata* and *S. marilandica* contained approximately 60.0% and 68.8% water, respectively (data not shown). As revealed in Figure 2, shading did not significantly affect the overall metabolite composition of either species. Although the shade and no shade plants clustered in the PCAs, significant differences between the metabolite profiles of May 2015 and July 2015 plants can be observed in Figure 2. The leaves of *S. lanceolata* plants grown without shade and harvested both in May 2015 and July 2015 had higher levels of harpagoside in comparison to shade-grown plants; however, these differences were only statistically different (*p* ≤ 0.05) for the July 2015 harvest (Figure 3). Contrarily, the leaves of *S. marilandica* harvested at the same times showed no differences in harpagoside content due to shading. *S. lanceolata* leaves not grown under shade and harvested in May 2015 produced 0.63 ± 0.08% harpagoside compared to shade-grown (0.43 ± 0.05%) plants (Figure 3). This species also produced more harpagoside in its leaves compared to *S. marilandica* (0.24 ± 0.04%) harvested at the same time. Yang, et al. [28] reported that ambient temperature positively correlated with harpagoside accumulation in *Scrophularia ningpoensis* Hemsl., roots, in which plants harvested in the summer contained the highest amount of harpagoside. Wang, et al. [26] found that this metabolite may protect *S. ningpoensis* roots from reactive oxygen species (ROS) during drought stress. In bioassays, harpagoside has antioxidant properties [30,31]. Apart from the decline in October 2014 and October 2015, harpagoside content in the roots of *S. lanceolata* and *S. marilandica* remained unchanged throughout the 2014 and 2015 growing seasons (Figure 4 and Figure 5). Because the plants in our study were irrigated, we can hypothesize (as with Wang, et al. [26]) that an increase in the production of harpagoside was not needed to mitigate the effects of drought.

Yang, et al. [28] found that environmental factors and seasonal variation did not significantly affect verbascoside content in *S. ningpoensis* roots. Alternatively, *Scrophularia striata* Boiss. plants subjected to drought stress have been reported to accumulate verbascoside in their roots [32]. The present study revealed that, after initially increasing in late spring (May 2014 and May 2015), verbascoside concentrations in *S. lanceolata* and *S. marilandica* roots were mostly unchanged (except for the October 2014 decline in *S. marilandica* roots) across the growing seasons (Figure 4 and Figure 5). Assuming that *S. lanceolata* and *S. marilandica* respond similarly to drought stress, these results reaffirm that the study plants were not under drought stress. The leaves and stems of both species accumulated verbascoside over the summer (Figure 4 and Figure 5). These results imply that seasonal variation or other environmental factors may influence the accumulation of verbascoside in the aerial tissues of *S. lanceolata* and *S. marilandica*.

Since *S. lanceolata* is most often found growing in semi-shaded areas in nature, it may be sensitive to ultraviolet-B (UV-B) radiation; possibly explaining why harpagoside content was higher in the no shade leaves (Figure 3). Plants exposed to UV-B can form ROS that damage carbohydrates, lipids, nucleic acids, and proteins. Increased production of antioxidant metabolites quenches ROS produced by UV-B damage [33]. In fact, in vitro *Scrophularia takesimensis* Nakai plants accumulated more harpagoside when grown under blue LED compared to white fluorescent light or red LED [22]. Those authors concluded that light quality significantly impacted harpagoside production.

Plants of both species grown without shade accumulated harpagide across the growing seasons (Figure 4 and Figure 5). In both May 2015 and July 2015, there were no differences in verbascoside content between the no shade and shade-grown plants of either species (Figure 4 and Figure 5); thus, verbascoside may not be involved with protecting the aerial tissues from UV-B damage. Londono, et al. [34] found similar results in *Primula veris* L. leaves and flowers. Different levels of UV radiation had no effect on total antioxidant content; however, particular antioxidants increased in response to greater UV-B exposure [34]. This suggests that some metabolites (such as harpagide and harpagoside) might be more important than other compounds in protecting against UV-B damage, especially in *S. lanceolata*. Verbascoside is a known antioxidant in bioassays [35], but this metabolite, as previously mentioned, may instead protect the aerial tissues from temperature stress during late summer growth.

Liang, et al. [24] observed that high temperature affected the iridoid glycoside content in *S. ningpoensis* roots during flowering. The plants subjected to high temperatures (45/38 °C [day/night]) accumulated less harpagide and harpagoside compared to control plants (28/23 °C [day/night]), and it was concluded that heat stress might inhibit the production of iridoid glycosides, especially harpagoside [24]. Across both growing seasons (2014 and 2015), harpagoside content declined in the leaves, stems, and roots of *S. marilandica* (Figure 5). Harpagide content remained unchanged (2014 season) or accumulated (2015 season) in the roots of *S. marilandica*, while the production of this metabolite decreased in the stems (except for the 2015 season) and leaves of this species (Figure 5). Similarly, both Li, et al. [23] and Xie, et al. [27] found that, in *S. ningpoensis*, harpagide in the stems and harpagoside in the leaves peaked in the summer and decreased thereafter. They hypothesized these changes were due to seasonal variation and/or temperature. Besides the decline in harpagide content in the stems of *S. lanceolata* plants grown during the 2014 season, this metabolite accumulated in the leaves, stems, and roots of this species throughout both seasons. The production of harpagoside did decline in the leaves of *S. lanceolata* in August 2014 but, in general, this metabolite accumulated in the leaves and stems of this species (Figure 4). As with both Li, et al. [23] and Xie, et al. [27], our results indicate that the production of harpagide and harpagoside in *S. marilandica* (like *S. ningpoensis*) may be sensitive to temperature or developmental changes.

The stems of *S. lanceolata* and *S. marilandica* species contained the highest percentage of harpagide [8,15]. According to Georgiev, et al. [18], harpagide may be transported throughout plants for the biosynthesis of harpagoside and harpagoside-related metabolites. The accumulation of harpagide in the stems of *S. lanceolata* and *S. marilandica* may also be involved in plant defense. Gowan, et al. [36] found that the iridoid glycoside, antirrhinoside, was transported in the phloem of *Maurandya scandens* (Cav.) Pers. [synonym: *Asarina scandens* (Cav.) Pennell] to potentially deter phloem-feeding pests. Antirrhinoside has a similar structure to harpagide. Harpagide has been shown to inhibit DNA polymerase in red flour beetles [37] and, like antirrhinoside, harpagide may protect against generalist insect herbivores and/or phloem-feeders.

## 3. Conclusions

This is the first study comparing the metabolite composition of *Scrophularia* species cultivated in the field subjected to different light intensities using a shade cloth. The optimal production of various metabolites that have important pharmacological applications was determined. The concentration of harpagoside in no shade *S. lanceolata* plants was greater than in shade-grown plants (Figure 3). Furthermore, the ability of *S. lanceolata* to produce more harpagoside and verbascoside in the field compared to *S. marilandica* may make this species a potential alternative to *H. procumbens* for use in dietary supplements. The ease of cultivation and harvesting leaves (instead of destructively harvesting roots or tubers) provides further support for the medicinal use of *S. lanceolata*. Nonetheless, the leaves of *S. lanceolata* have not been tested in bioassays. Studies comparing *S. lanceolata* leaf extracts to *H. procumbens* tuber extracts could determine the relative bioactivity and safety of this species.

## 4. Materials and Methods

Significant details on site (climate, location, site preparation, and soil) and plants used (seed source, germination protocol, greenhouse production, and transplanting) for this study are provided in Brownstein, et al. [8]. Briefly, the site in southwest Missouri was established on 25 June 2013 by transplanting 900 seedlings (450 of each *Scrophularia* species) into a pre-prepared site (approximately 40 × 4 m) that incorporated drip irrigation and a woven weed barrier fabric for weed control. Ten field plots (≈ 3.4 × 3.0 m) were established in a side-by-side, east–west orientation. Each plot included four east–west data rows of eight plants each. Each data row was flanked on both sides by border rows of similar plants with row ends also buttressed by border plants so that all study plants were grown in near-identical conditions, even as some plants were harvested. Thus, each plot contained nine rows of ten plants each (90 plants per plot). The two *Scrophularia* species were each randomly assigned to two of the four data rows in each plot. Five of the plots remained open to full sun, while five were randomly assigned shade as a treatment. Individual wooden shade structures (approximately 3.5 m high) were built over designated plots onto which black, woven polypropylene shade cloth that excluded 40% of sunlight (PAK Global, Cornelia, GA, USA) was attached on 16 May 2014 for the duration of the study. Because the plots were located in an east–west orientation, the shade cloth covered the top and south facing side of each structure. In southwest Missouri, east–west plots are ideal because north–south rows lead to uneven shading. All plots were irrigated simultaneously to provide about 2.5 cm of water per week when rainfall was lacking during the growing seasons.

After the Brownstein, et al. [8] study (concluded May 2014) and after shade treatments were imposed, we harvested seven sample sets over two growing seasons. In 2014, plants were harvested during active spring growth and anthesis (May and June), at completion of active seasonal growth with full fruit ripeness (August), and at autumn dormancy (October). In 2015, plants were surveyed while in active spring growth and anthesis (May), full plant maturity with fruit ripening (July), and autumn dormancy (October). For each harvest, one randomly selected plant per species from each of the ten plots was excavated and plant morphology (plant height, inflorescence height, and stem number) was determined. Specimens were then washed of all soil, after which leaves, stems, and roots were separated. For the October 2014 and October 2015 harvests, the plants had mostly senesced for the season; therefore, only the roots were collected. After harvesting, the samples were immediately dried for 2 days at ≈40 °C in a ventilated propane-fired dryer, after which they were ground and stored at −20 °C pending laboratory analysis.

In 2016, 40 mg of leaf, stem, and root samples were extracted with 1.0 mL of cold methanol (4 °C). The samples were sonicated continuously in a cold-water bath (4 °C) for 2 h and then centrifuged at 5000× *g* for 10 min at 4 °C. The supernatant (0.10 mL) was transferred to a new microtube and diluted with 0.40 mL of cold water-acetonitrile [1:1]/0.10% formic acid (4 °C). This solution was filtered through a 0.20 μm filter into a sample vial and analyzed on a Waters Acquity ultra-performance liquid chromatography (UPLC) system with a photodiode array (PDA) detector coupled to a Waters Synapt G2-S (Waters Corporation, Milford, MA, USA) mass spectrometry (MS) instrument following the UPLC-MS parameters described in Brownstein, et al. [15].

Verbascoside and harpagide were purchased from PhytoLab GmbH & Company KG (Vestenbergsgreuth, Germany), and harpagoside was purchased from ChromaDex Corporation (Los Angeles, CA, USA). Under the present UPLC-MS conditions, harpagide (363.13 [M-H]^−^ *m*/*z*), harpagoside (493.17 [M-H]^−^ *m*/*z*), and verbascoside (623.19 [M-H]^−^ *m*/*z*) eluted at 1.26, 5.61, and 3.67 min, respectively. From the PDA raw data, a 7-point standard curve was developed for harpagoside at 280 nm. The peak areas of harpagoside were processed in TargetLynx (Waters Corporation, Milford, MA, USA). For principal component analysis (PCA), the datasets were processed in Progenesis QI (Nonlinear Dynamics, Newcastle upon Tyne, UK) using the following parameters: the automatic sensitivity method value was set at 1 (fewer); peak widths at and less than 0.19 min were ignored; ions after 12 min were ignored; and the samples were normalized to all compounds. With these parameters, about 1500 mass spectral features were detected. Statistical significance (*p* ≤ 0.05) was determined by independent/unpaired two-sample *t*-test or Wilcoxon test using R programming language.

## Data Availability

The data presented in this study are available in the article and supplementary materials. Additional data, such as mass spectral raw folders, are available on request from the corresponding authors.

## Supplementary Materials

The following are available online at https://www.mdpi.com/article/10.3390/metabo11070464/s1, Figure S1: Relative abundances (low [0.00] to high [1.00] of harpagide (A,D,G,J), harpagoside (B,E,H,K), and verbascoside (C,F,I,L) in leaves (A–C), stems (D–F), and roots (G–I) of *S. lanceolate* plants over the growing season (A–I) and under shade and no shade treatments (J–L)), Figure S2: Relative abundances (low [0.00] to high [1.00] of harpagide (A,D,G,J), harpagoside (B,E,H,K), and verbascoside (C,F,I,L) in leaves (A–C), stems (D–F), and roots (G–I) of *S. marilandica* plants over the growing season (A–I) and under shade and no shade treatments (J–L)), Table S1: Maximum (Max) and minimum (Min) ambient temperatures, total annual precipitation, and growing degree days (base 50 °F) recorded at or near the Scrophularia study, 2014–2015, Mt. Vernon, MO.

## References

[1] Scheunert A., Heubl G. (2011). Phylogenetic Relationships among New World *Scrophularia* L. (Scrophulariaceae): New insights inferred from DNA sequence data. Plant Syst. Evol..

[2] de Galindez J.S., Lanza A.M.D., Fernandez L.M. (2002). Biologically Active Substances from the Genus *Scrophularia*. Pharm. Biol..

[3] Garcia D., Fernandez A., Saenz T., Ahumada C. (1996). Anti-inflammatory Effects of Different Extracts and Harpagoside Isolated from *Scrophularia frutescens* L. Farmaco.

[4] Tundis R., Loizzo M.R., Menichini F., Statti G.A., Menichini F. (2008). Biological and pharmacological Activities of Iridoids: Recent Developments. Mini Rev. Med. Chem..

[5] Herrick J.W. (1997). Iroquois Medical Botany. Ph.D. Thesis.

[6] Hough F.B. (1849). The medicinal qualities of *Scrophularia marilandica*. N. Engl. J. Med..

[7] Moerman D. (1998). Native American Ethnobotany.

[8] Brownstein K.J., Thomas A.L., Rottinghaus G.E., Lynch B.A., Gang D.R., Folk W.R. (2016). Harpagide and related iridoid glycosides in vegetative tissues of cultivated *Scrophularia lanceolata* and *Scrophularia marilandica*. Acta Hortic..

[9] Stewart K.M., Cole D. (2005). The commercial harvest of devil’s claw (*Harpagophytum* spp.) in southern Africa: The devil’s in the details. J. Ethnopharmacol..

[10] Gagnier J.J., Oltean H., van Tulder M.W., Berman B.M., Bombardier C., Robbins C.B. (2016). Herbal medicine for low back pain: A Cochrane review. Spine.

[11] Mncwangi N., Chen W., Vermaak I., Viljoen A.M., Gericke N. (2012). Devil’s claw: A review of the ethnobotany, phytochemistry and biological activity of *Harpagophytum procumbens*. J. Ethnopharmacol..

[12] Oltean H., Robbins C., van Tulder M.W., Berman B.M., Bombardier C., Gagnier J.J. (2014). Herbal medicine for low-back pain. Cochrane Database Syst. Rev..

[13] Ungerer G., Cui J., Ndam T., Bekemeier M., Song H., Li R., Siedhoff H.R., Yang B., Appenteng M.K., Greenlief C.M. (2020). *Harpagophytum procumbens* extract ameliorates allodynia and modulates oxidative and antioxidant stress pathways in a rat model of spinal cord injury. Neuromol. Med..

[14] Schneider E., Sanders J., Von Willert D. (2006). Devil’s claw (*Harpagophytum procumbens*) from Southern Africa: Sustainable use by cultivation combined with controlled harvesting in semi-wild populations. Frontis.

[15] Brownstein K.J., Gargouri M., Folk W.R., Gang D.R. (2017). Iridoid and phenylethanoid/phenylpropanoid metabolite profiles of *Scrophularia* and *Verbascum* species used medicinally in North America. Metabolomics.

[16] Abdelouahab N., Heard C. (2008). Effect of the major glycosides of *Harpagophytum procumbens* (devil’s claw) on epidermal cyclooxygenase-2 (COX-2) in vitro. J. Nat. Prod..

[17] Anauate M.C., Torres L.M., de Mello S.B.V. (2010). Effect of isolated fractions of *Harpagophytum procumbens* DC (devil’s claw) on COX-1, COX-2 activity and nitric oxide production on whole-blood assay. Phytother. Res..

[18] Georgiev M.I., Ivanovska N., Alipieva K., Dimitrova P., Verpoorte R. (2013). Harpagoside: From Kalahari Desert to pharmacy shelf. Phytochemistry.

[19] Gyurkovska V., Alipieva K., Maciuk A., Dimitrova P., Ivanovska N., Haas C., Bley T., Georgiev M. (2011). Anti-inflammatory activity of devil’s claw in vitro systems and their active constituents. Food Chem..

[20] Viljoen A., Mncwangi N., Vermaak I. (2012). Anti-inflammatory iridoids of botanical origin. Curr. Med. Chem..

[21] de Galindez J.S., Matellano L.F., Lanza A.M.D., Castillo L.V. (2000). Seasonal variation in the harpagoside content of *Scrophularia scorodonia* L. Z. Naturforsch. C J. Biosci..

[22] Jeong B.R., Sivanesan I. (2015). Direct adventitious shoot regeneration, in vitro flowering, fruiting, secondary metabolite content and antioxidant activity of *Scrophularia takesimensis* Nakai. Plant Cell Tissue Organ Cult..

[23] Li H.W., Liu P., Zhang H.Q., Feng W.M., Yan H., Guo S., Qian D.W., Duan J.A. (2020). Determination of bioactive compounds in the nonmedicinal parts of *Scrophularia ningpoensis* using ultra-high-performance liquid chromatography coupled with tandem mass spectrometry and chemometric analysis. J. Sep. Sci..

[24] Liang Z.S., An Y.Y., Liu H.Y. (2014). High temperature stress decreases root iridoid glycosides biosynthesis of *Scrophularia ningpoensis* during florescence. J. Med. Plant Res..

[25] Mncwangi N.P., Viljoen A.M., Zhao J., Vermaak I., Chen W., Khan I. (2014). What the devil is in your phytomedicine? Exploring species substitution in *Harpagophytum* through chemometric modeling of 1 H-NMR and UHPLC-MS datasets. Phytochemistry.

[26] Wang D.H., Du F., Liu H.Y., Liang Z.S. (2010). Drought stress increases iridoid glycosides biosynthesis in the roots of *Scrophularia ningpoensis* seedlings. J. Med. Plant Res..

[27] Xie G., Jiang Y., Huang M., Zhu Y., Wu G., Qin M. (2020). Dynamic analysis of secondary metabolites in various parts of *Scrophularia ningpoensis* by liquid chromatography tandem mass spectrometry. J. Pharm. Biomed. Anal..

[28] Yang S., Li J., Zhao Y., Chen B., Fu C. (2011). Harpagoside variation is positively correlated with temperature in *Scrophularia ningpoensis* Hemsl. J. Agric. Food Chem..

[29] National Oceanic and Atmospheric Administration (NOAA) North American Climate Extremes Monitoring. https://www.ncdc.noaa.gov/extremes/nacem/methodology.

[30] Georgiev M.I., Alipieva K.I., Denev P. (2010). Antioxidant activity and bioactive constituents of the aerial parts of *Harpagophytum procumbens* plants. Biotechnol. Biotechnol. Equip..

[31] Jeong E.J., Lee K.Y., Kim S.H., Sung S.H., Kim Y.C. (2008). Cognitive-enhancing and antioxidant activities of iridoid glycosides from *Scrophularia buergeriana* in scopolamine-treated mice. Eur. J. Pharmacol..

[32] Falahi H., Sharifi M., Maivan H.Z., Chashmi N.A. (2018). Phenylethanoid glycosides accumulation in roots of *Scrophularia striata* as a response to water stress. Environ. Exp. Bot..

[33] Blokhina O., Virolainen E., Fagerstedt K.V. (2003). Antioxidants, oxidative damage and oxygen deprivation stress: A review. Ann. Bot..

[34] Londono P.T., Papagiannopoulos M., Gobbo-Neto L., Muller C. (2014). Variation in flavonoid pattern in leaves and flowers of *Primula veris* of different origin and impact of UV-B. Biochem. Syst. Ecol..

[35] Alipieva K.I., Korkina L., Orhan I.E., Georgiev M.I. (2014). Verbascoside: A review of its occurrence, (bio)synthesis and pharmacological significance. Biotechnol. Adv..

[36] Gowan E., Lewis B.A., Turgeon R. (1995). Phloem transport of antirrhinoside, an iridoid glycoside, in *Asarina scandens* (Scrophulariaceae). J. Chem. Ecol..

[37] Pungitore C.R., Ayub M.J., Garcia M., Borkowski E.J., Sosa M.E., Ciuffo G., Giordano O.S., Tonn C.E. (2004). Iridoids as allelochemicals and DNA polymerase inhibitors. J. Nat. Prod..

