# Supplementary figures and images for: Changes in the Harpagide, Harpagoside, and Verbascoside Content of Field Grown Scrophularia lanceolata and Scrophularia marilandica in Response to Season and Shade
Source: Metabolites. 2021 Jul 19;11(7):464. doi: 10.3390/metabo11070464 (PMC8308087; doi:10.3390/metabo11070464)

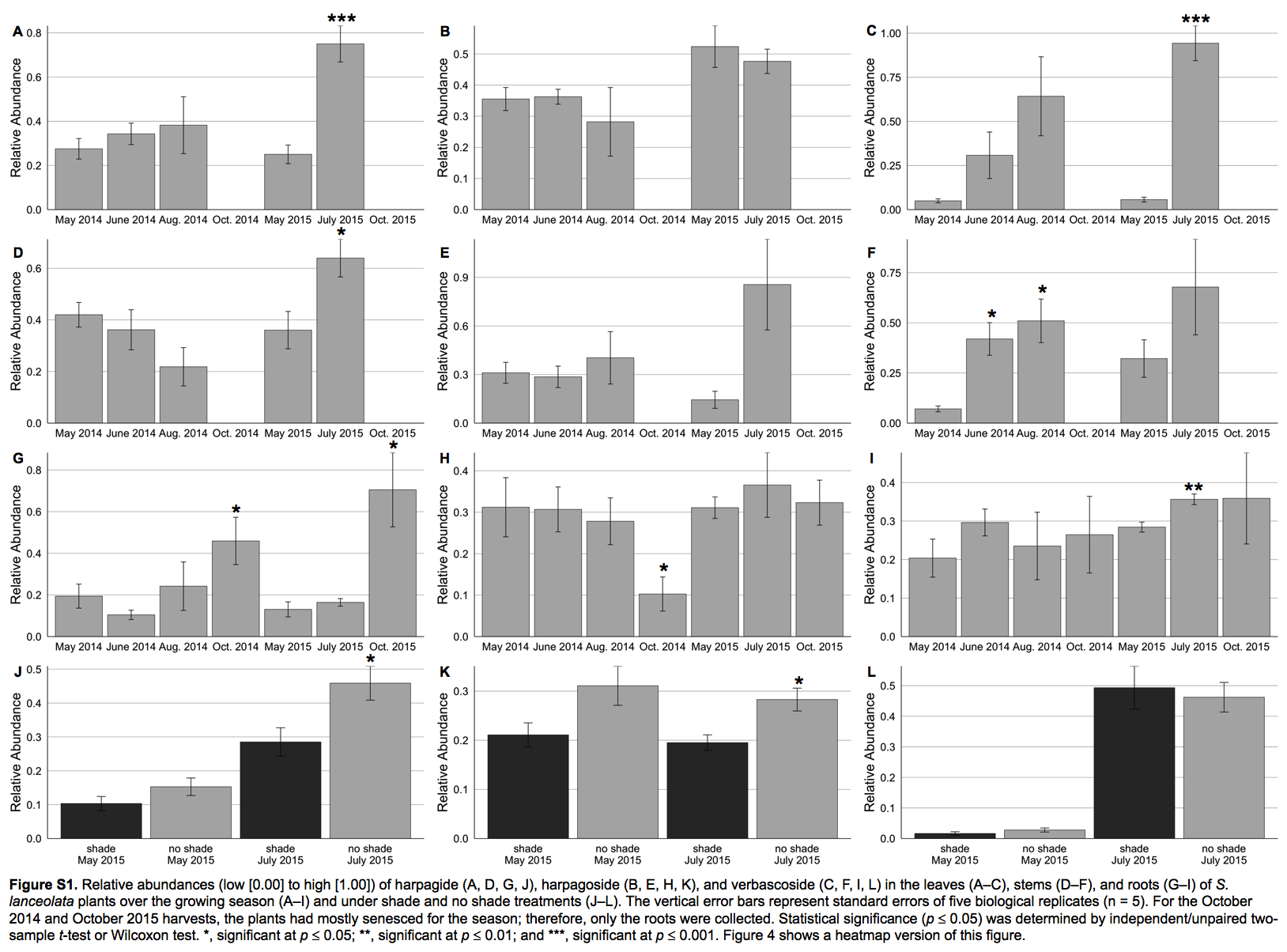

Supplement: Supplementary file 1 [file metabolites-11-00464-s001.zip › Figure_S1.tif]

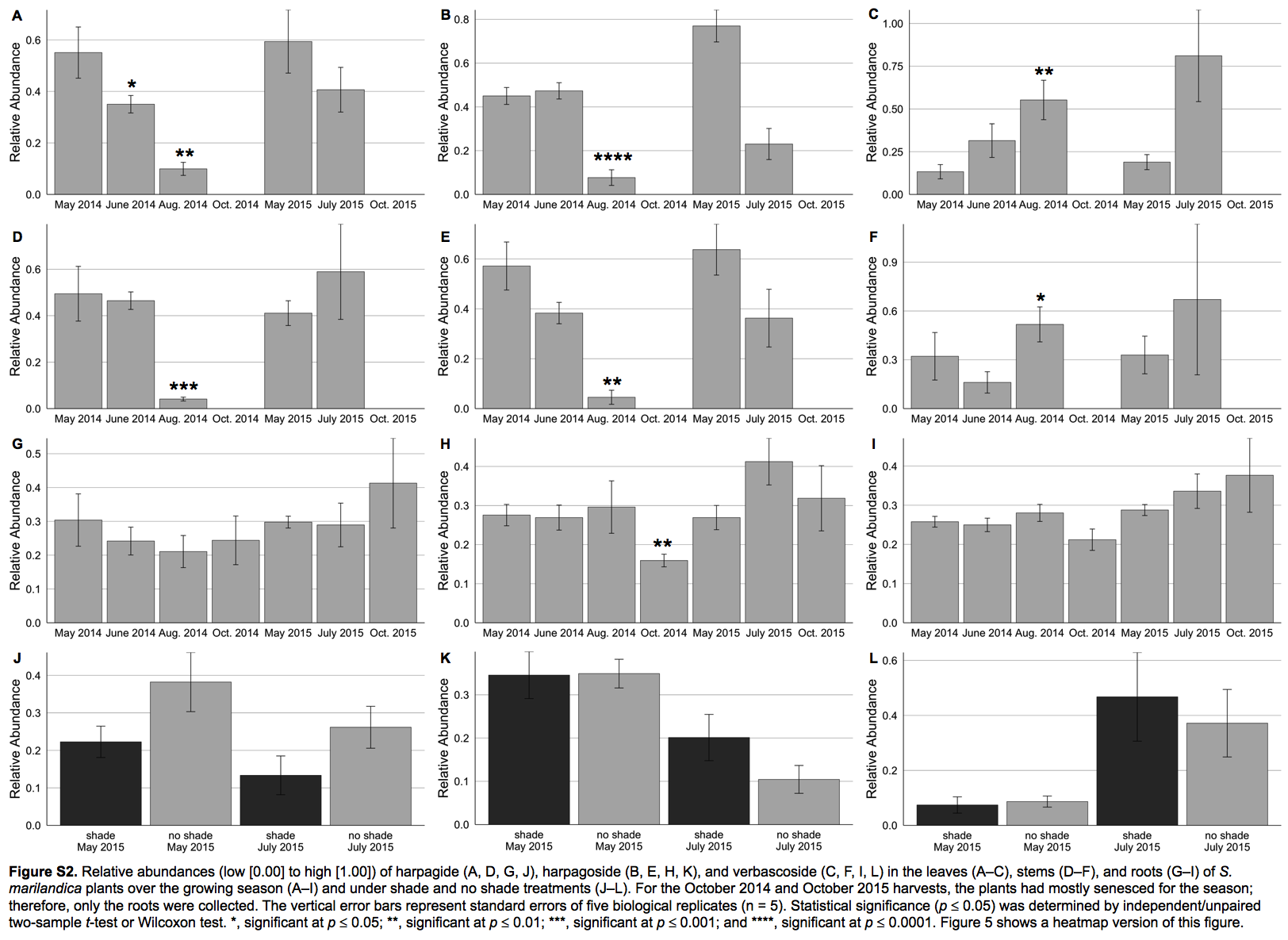

Supplement: Supplementary file 1 [file metabolites-11-00464-s001.zip › Figure_S2.tif]
